# Mobile Health Adoption in High-Risk Pregnancies Using Cluster Analysis of Biopsychosocial Outcomes: Observational Longitudinal Cohort Study

**DOI:** 10.2196/67680

**Published:** 2025-08-21

**Authors:** Fernanda Schier de Fraga, Mayara Marenda Narita, Monique Schreiner, Flavio Belli, Jaqueline Leonel Celestino, Karolayne Braz Pereira, Gabriella Soecki, Vitória Bevervanso, Rogério de Fraga

**Affiliations:** 1 Department of Obstetrics and Gynecology of the Federal University of Paraná, Rua General Carneiro, 181, Curitiba, 80060-900, Brazil, 55 41991213082; 2 Department of Surgical Clinics at the Federal University of Paraná, Curitiba, Brazil

**Keywords:** antenatal care, cluster analysis, mHealth, mobile health, high risk, pregnancy, biopsychosocial, mobile technology, high-risk pregnancy, self-management, pregnant, antenatal, mobile health app, health perception, app, observational longitudinal cohort study, Brazil, smartphone, mobile phone

## Abstract

**Background:**

The use of mobile technologies during high-risk pregnancy, placing patients at the center of care, affords them self-management and easier access to health information.

**Objective:**

This study aims to understand the health perception of pregnant women at the beginning of high-risk antenatal care, the usability of a mobile health app—the Health Assistant—and to compare maternal-fetal outcomes between users and nonusers of the app.

**Methods:**

This is an observational longitudinal cohort study that looked into clusters of high-risk pregnant women admitted to antenatal care at the maternity unit of a public university hospital in southern Brazil between April 2022 and November 2023. Pregnant women who did not have a compatible smartphone to download the app or who did not have internet access were excluded from the study. According to systematic randomization, one patient was allocated to the app group and the other to the control group. They all answered an inclusion questionnaire (Q1), and those in the app group were instructed to use the Health Assistant app to prepare for their first antenatal appointment, which would take place in a few weeks’ time, when they would answer the Brazilian version of the Mobile App Usability Questionnaire. After childbirth, maternal-fetal outcomes were assessed. Student 2-tailed *t* test, Mann-Whitney test, Fisher exact test, and the chi-square test were used for statistical analysis. A hierarchical cluster analysis was performed using the Ward method and the Euclidean squared distance measure.

**Results:**

The sample contained 111 pregnant women, of whom 55 (49.5%) were allocated to the app group and 56 (50.5%) to the control group. Of the 55 pregnant women who used the app, 21 (38.2%) demonstrated adherence, with an average Mobile App Usability Questionnaire score of 6.2 (SD 1.0). Clustering included 110 pregnant women, and the dendrogram resulted in three clusters, which show several significant differences in terms of family income, medical history, medication adherence, and lifestyle habits. Cluster 2 had the lowest adherence to the app (*P*=.08) and attended significantly fewer antenatal appointments (6.9 appointments) as compared with Clusters 1 (10.3) and 3 (9.1; *P*=.006). Cesarean section was more frequent in Cluster 3 (n=41, 95.3%) as compared with Clusters 1 (n=12, 27.9%) and 2 (n=5, 20.8%), *P*<.001.

**Conclusions:**

Cluster analysis, revealing different profiles of pregnant women, allowed us to identify groups that would benefit from personalized approaches and digital interventions to improve self-awareness and gestational outcomes. The Health Assistant app showed good usability in this context.

## Introduction

Mobile health (mHealth) is defined as medical and public health practice aided by mobile devices such as cellphones, patient monitoring tools, personal digital assistants, and other wireless equipment [1,2]. In Brazil, smartphone price reduction and improved connectivity facilitating access to device functionalities have had a major impact on health care with useful tools for professionals and patients alike [3,4]. As part of patient care, mHealth apps aim to promote well-being, emotional, psychological, and physical growth, to increase accessibility to health services in remote areas, and to make processes faster and more precise [2,4-6]. The use of mHealth technologies during pregnancy and the postpartum period has become more common with the advance of information and communication technologies [7,8].

Despite the great educational potential and the increasing popularity of health apps, rigorous scientific evaluation of their effectiveness is still limited, and there is an insufficient number of studies using standardized methods to guarantee their performance and safety [8,9]. DeNicola et al [8] conclude that these apps should be developed with the cooperation of health professionals. An ideal app, resting on scientific evidence, respecting the law, and protecting privacy, should also be user-friendly, attractive, simple, and functional, deploying device sensors and resources for the good of the patient [10]. The use of this technology can thus prove a valuable strategy for health self-management, transforming patient experience by affording greater autonomy and ease of access to care [11].

High-risk pregnancy—when medical conditions or specific pregnancy factors can compromise the health of the mother or the fetus—has an approximate prevalence of 22% [12,13]. This condition can intensify stress and impact the psychological health of pregnant women, worried about possible interventions, hospitalizations, and treatments [14]. The very label “high-risk pregnancy” can contribute to additional stress, as concerns may focus rather on treatments and tests than on the woman’s overall well-being, but it should also lead to greater medical vigilance, which can be perceived either positively or negatively by the patient [12].

In these cases, patient-centered communication yields positive results, since a more humanized and integrative approach can significantly improve the quality of care [15]. Several studies show that a person-centered approach improves satisfaction, reduces costs, and increases the effectiveness of treatments, especially in patients with chronic conditions [15]. In this context, the use of mobile technologies places the patient at the center of care, offering a new experience that enables health self-management and easier access to information and services [11]. A systematic review identified that in 74.3% of the studies, mHealth intervention was cost-effective, but indicated that more research is needed in low- and lower-middle-income countries for the impact of different types of mHealth to be better understood [16].

Furthermore, cluster analysis, considered a relevant method of classifying women in early pregnancy, is another way of drawing attention to the follow-up of high-risk pregnant patients [17]. By identifying homogeneous groups, it allows risk factors to be detected, as well as patterns and trends in pregnancy outcomes to be understood [18,19].

Since longitudinal follow-up with no information gaps is essential for proper antenatal care, an mHealth app produced by health professionals could help prepare high-risk pregnant women for their first antenatal appointment at a maternity unit of a public university hospital in southern Brazil. An easy-to-use app called Health Assistant was expected to have positive effects on both the pregnant woman and the child.

The aim of this study was to evaluate the health perception of pregnant women starting high-risk antenatal care in a low-income country, the usability of the Health Assistant, and maternal-fetal outcomes among app users and nonusers so that we could identify those groups of patients who benefit most from the app and thus provide focused assistance in order to optimize care and obstetric outcomes.

## Methods

### Recruitment

Pregnant women were included upon admission to high-risk antenatal care at the maternity unit of a public university hospital in southern Brazil between April 2022 and November 2023. High-risk pregnancies were characterized by chronic conditions or those diagnosed during pregnancy, such as hypertension, gestational diabetes, and mood disorders. Primary care referral was the gateway to this care.

Pregnant women who did not have a compatible smartphone to download the app or who did not have internet access, even when the researchers provided a router, were excluded from the study. After accepting and signing the informed consent form, participants were included in the study and allocated, according to systematic randomization with random start, to one of the groups: app or control. The first patient was drawn at random and allocated to the app group, and the others were then assigned in an interleaved manner: the second to the control group, the third to the app group, the fourth to the control group, and so on. Thus, we created two balanced groups—one invited to use the app and the other not invited—without supervision or imposition of use. Although the allocation was random, the study maintained an observational design, as the actual use of the app was at the discretion of the participants and there was no controlled intervention beyond the invitation.

Every patient answered the inclusion questionnaire (Q1), consisting of 32 questions about their social, economic, and health profile. Those allocated to the app group were instructed to use the Health Assistant app to prepare for their first antenatal appointment, which would take place in a few weeks’ time. The digital tool was used without supervision or direct control by the researchers and allowed patients to answer detailed questions about their current pregnancy and their gynecologic-obstetric history. Our objective was to evaluate, in the context of routine clinical practice, the associations between digital engagement and maternal-fetal outcomes, and not to test a controlled intervention protocol.

At the first antenatal appointment, researchers met with participants from the app group and had adherents to the app answer the Brazilian version of the Mobile App Usability Questionnaire (MAUQ).

### Ethical Considerations

This is an observational longitudinal cohort study approved by the Institutional Ethics Committee of the Hospital de Clínicas Complex (47084021.5.0000.0096). All participants provided written informed consent before enrollment in the study. Participants’ personal data were anonymized to preserve confidentiality and privacy. No financial or other compensation was offered for participation.

### MAUQ

MAUQ was chosen because it is a widely recognized tool in the literature for evaluating the usability of mHealth apps. Developed and validated in English by Zhou et al [20], the MAUQ is highly reliable with a Cronbach α of 0.914. The questionnaire was translated into Portuguese by Machado et al in accordance with the guidelines for translation and cross-cultural adaptation of nontechnical skills assessment instruments, ensuring similarity between the original and the translation. Permission to use the translation was kindly granted to the researchers (personal communication by Machado, 2022). For this study, we used the version of the MAUQ for stand-alone digital health apps intended for patients to control, manage, and improve their health care. The questionnaire assessed the following parameters: ease of use, interface, satisfaction, and usefulness. Participants rated each item using a 7-point Likert Scale, ranging from 1=strongly disagree to 7=strongly agree. The usability of the app was determined by the total score and the averages of all the questionnaires answered, where a higher score indicates greater usability [20]. In our study, the MAUQ was administered to patients who were considered adherent, that is, who reported having used the app.

### Outcome Analysis

After the expected date of delivery, researchers retrieved their medical records to analyze maternal-fetal outcomes, including the number of antenatal appointments, the number of emergency room visits during antenatal care, intercurrent events, date of delivery, route of delivery, complications, and length of stay for mother and baby, among others. The information collected was organized and filed in spreadsheets for statistical analysis.

### Cluster Analysis

Clustering is a technique for organizing a large amount of information into smaller groups so that hidden patterns and relationships between the data are identified and personalized interventions are developed [21,22]. This method was chosen because of its effectiveness in identifying the hierarchical structure of binary data without the need to specify the number of desired clusters beforehand. It also promotes greater homogeneity within clusters and is easy to replicate, making it applicable to various types of data [21,23,24]. Based on the characteristics and outcomes of each cluster, it is possible to develop antenatal care strategies that optimize patient-centered care.

### Sample Size Calculation

As an exploratory technique that does not require statistical hypotheses, hierarchical cluster analysis generally dispenses with sample calculation, since there are no standardized methods for it [25]. Dalmaijer et al [26] suggest that a sample of 20‐30 individuals per expected subgroup is sufficient for cluster analysis, provided that there is a clear separation between the subgroups. Defining the sample in this study aimed to ensure that there was enough data for significant patterns to emerge. Estimating the formation of two or three clusters, depending on the dendrogram, a minimum of 100 patients was therefore proposed, 50 for the control group and 50 for the app group.

### Statistical Analysis

Data were organized in an Excel (Microsoft Corp) spreadsheet and analyzed using SPSS Statistics (version 29.0.0; IBM Corp). Descriptive analysis of quantitative variables included the mean, SD, median, and IQR. Categorical variables were expressed in frequency and percentage. Student 2-tailed *t* test for independent samples or the Mann-Whitney nonparametric test was used to compare control and app groups in terms of quantitative variables. The normality of continuous variables was assessed using the Kolmogorov-Smirnov test. Categorical variables were analyzed using Fisher exact test or the chi-square test. An exploratory analysis of the variables in the inclusion questionnaire (Q1) resulted in the exclusion of questions with well-correlated answers or questions that had five or more missing data (blank answers). To determine the clustering of pregnant women, a hierarchical cluster analysis was carried out using the Ward method and the squared Euclidean distance measure. The number of clusters was determined according to the dendrogram. Values of *P*<.05 indicated statistical significance.

## Results

### Overview

**Figure 1.**
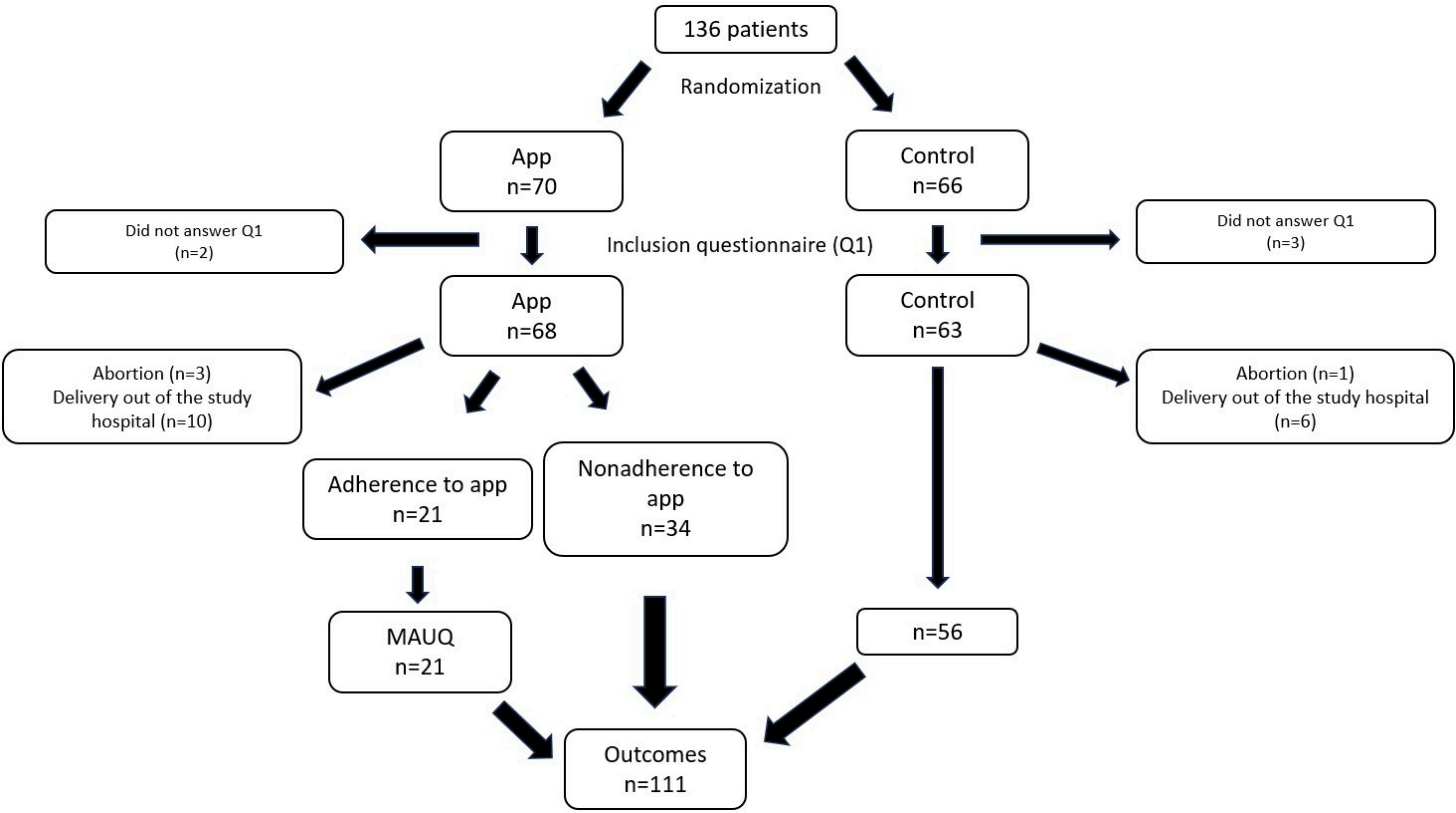
Patient inclusion flowchart. MAUQ: Mobile App Usability Questionnaire; Q1: inclusion questionnaire.

The inclusion questionnaire (Q1) was answered by 131 patients. The team had no access to the outcomes of 20 patients due to abortion or delivery out of the study hospital. Only patients who had answered Q1 and whose outcomes were available were included in the analysis, resulting in a total of 111 pregnant women, as shown in Figure 1.

The MAUQ, restricted to the group that was adherent to the app, was answered by 21 patients and had an average score of 6.2 (SD 1.0) points, with a median of 6.6 (IQR 2.3-7). The internal consistency of the instrument was high, with a Cronbach α estimated at 0.94.

### Patient Characteristics

The average age in the control group was 30.5 (SD 5.7) years and the app group was 28.8 (SD 6.2) years, with no significant difference (*P*=.13). The median number of pregnancies for the control group was 3 (IQR 1-10) and 2 (IQR 0‐6) for the app group (*P*=.18). Table 1 shows the categorical variables of Q1 with the characteristics of patients for each group.

**Table 1.** The inclusion questionnaire (Q1) categorical variables.

Variable and classification	Group, n (%)		*P* value^a^
	Control	App	
Marital status (n=111)			—^b^
Cohabiting	10 (17.9)	19 (34.5)	
Married	32 (57.1)	19 (34.5)	
Single	13 (23.2)	17 (30.9)	
Separated	1 (1.8)	0 (0)	
What is your average family income (in minimum wages; n=111)			.81
Less than 1 or 1	10 (17.9)	11 (20)	
2 or more	46 (82.1)	44 (80)	
Parity (n=111)			.23
Nulliparous	16 (28.6)	22 (40)	
Multiparous	40 (71.4)	33 (60)	
Have you ever had a vaginal birth? (n=111)			≥.99
No	24 (42.9)	23 (41.8)	
Yes	32 (57.1)	32 (58.2)	
How many vaginal births? (if applicable; n=64)			.75
1 or 2	25 (78.1)	27 (84.4)	
3 to 7	7 (21.9)	5 (15.6)	
Have you ever had a C-section? (n=111)			.57
No	23 (41.1)	26 (47.3)	
Yes	33 (58.9)	29 (52.7)	
How many C-sections? (if applicable; n=62)			.09
1 or 2	22 (66.7)	25 (86.2)	
3, 4, or 5	11 (33.3)	4 (13.8)	
Do you have any deceased child? (n=111)			.18
No	51 (91.1)	45 (81.8)	
Yes	5 (8.9)	10 (18.2)	
Do you understand why you have been referred to high-risk antenatal care? (n=111)			.62
No	3 (5.4)	1 (1.8)	
Yes	53 (94.6)	54 (98.2)	
Comorbidities (n=111)			.70
Only 1	34 (60.7)	31 (56.4)	
More than 1	22 (39.3)	24 (43.6)	
Are you on any sort of medication? (n=111)			≥.99
No	11 (19.6)	11 (20)	
Yes	45 (80.4)	44 (80)	
Do you know what each medication is used for? (n=95)			.49
No	6 (12.2)	3 (6.5)	
Yes	43 (87.8)	43 (93.5)	
Do you often forget to take your medication? (n=95)			.48
No	38 (77.6)	32 (69.6)	
Yes	11 (22.4)	14 (30.4)	
Have you brought the tests carried out so far in your pregnancy? (n=111)			.56
No	8 (14.3)	5 (9.1)	
Yes	48 (85.7)	50 (90.9)	
Do you understand why you had these tests? (n=111)			.12
No	9 (16.1)	3 (5.5)	
Yes	47 (83.9)	52 (94.5)	
Do you smoke? (n=111)			.56
No	48 (85.7)	50 (90.9)	
Yes	8 (14.3)	5 (9.1)	
Do you drink alcohol? (n=111)			.68
No	52 (92.9)	53 (96.4)	
Yes	4 (7.1)	2 (3.6)	
How do you rate the quality of your sleep? (n=111)			.15
Bad (0)	14 (25)	7 (12.7)	
Regular or good	42 (75)	48 (87.3)	
How do you rate the quality of your food? (n=110)			.49
Bad (0)	6 (10.9)	3 (5.5)	
It can be better or it is good	49 (89.1)	52 (94.5)	
Do you consider yourself anxious? (n=111)			≥.99
No	5 (8.9)	5 (9.1)	
Yes	51 (91.1)	50 (90.9)	
Do you consider yourself to be self-aware about your health (ie, do you understand your health and what it takes to stay healthy)? (n=111)			.79
No	7 (12.5)	8 (14.5)	
Yes	49 (87.5)	47 (85.5)	
Do you consider yourself happy with your health? (n=111)			.26
No or moderately	27 (48.2)	33 (60)	
Yes or very much	29 (51.8)	22 (40)	
Do you consider preventive care an important aspect of health? (n=111)			.49
No	2 (3.6)	0 (0)	
Yes	54 (96.4)	55 (100)	

a Fisher exact test or chi-square test; significance set at *P*<.05.

b Not applicable.

### Cluster Analysis

**Figure 2.**
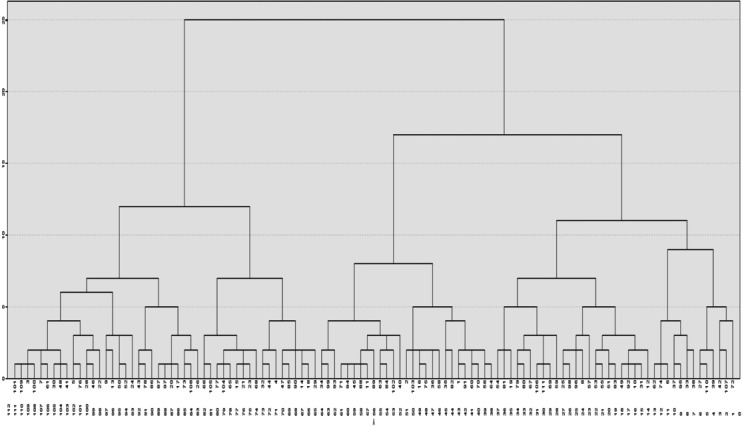
Dendrogram using Ward linkage—rescaled distance cluster combination.

Once it was determined which questions from Q1 would be included in the cluster analysis, one patient who did not adequately answer two or more of them ended up being excluded, which resulted in the analysis of data from 110 pregnant women. According to the dendrogram, the greatest leap in the Ward algorithm was from 86% to 93%, resulting in three groups of pregnant women (Figure 2).

Cluster 1 included 39.1% (n=43) of the participants, Cluster 2 had 21.8% (n=24), and Cluster 3 also included 39.1% (n=43). Pregnant women in Cluster 1 attended an average of 10.3 antenatal appointments, with a median of 11 (IQR 1-23). Participants in Cluster 2 had an average of 6.9 appointments, with a median of 6.5 (IQR 1-12). Finally, Cluster 3 showed an average of 9.1 antenatal appointments, with a median of 9 (IQR 1-24).

**Figure 3.**
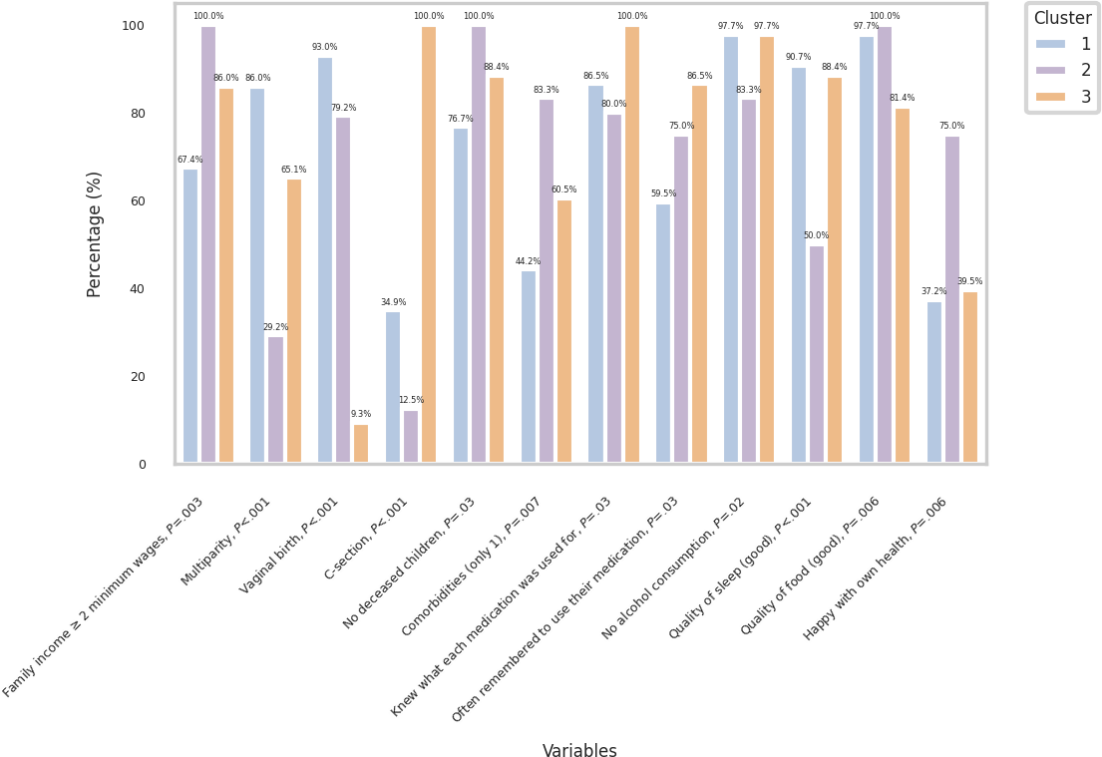
Variables per cluster.

**Figure 4.**
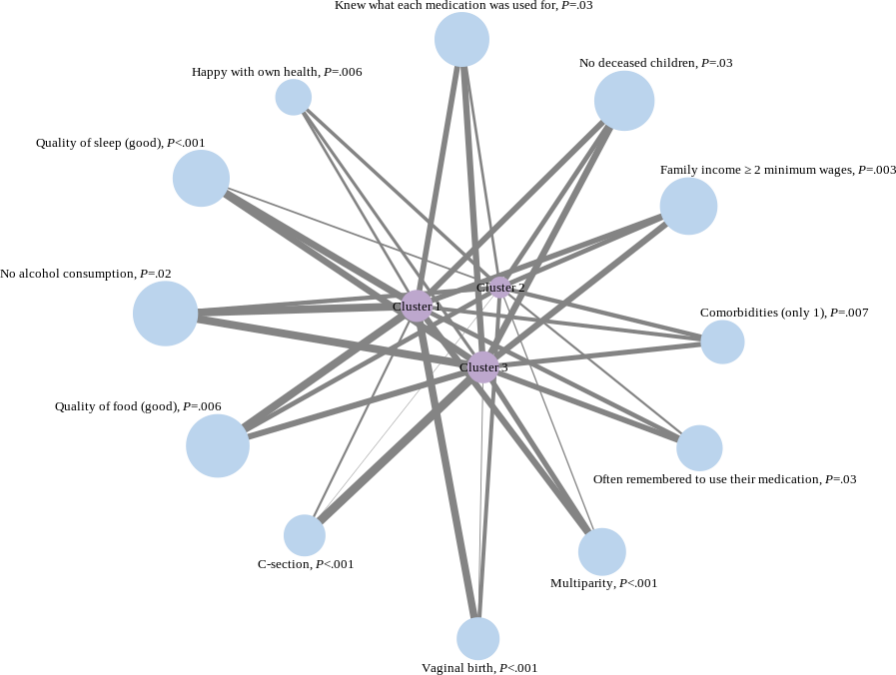
Nodes and connections per cluster.

Figure 3 shows how significant variables are distributed among clusters, based on the positively significant answers to Q1. A visual representation of the groups in nodes and their connections was created to show the associations between the different variables within clusters (Figure 4).

Acceptance and use of the app varied between the different clusters, with Cluster 1 showing the highest adherence and Cluster 2 the lowest (*P*=.08). Table 2 shows the differences in adherence to the app among clusters, including participants in the control group.

The outcomes that stood out among clusters were the number of antenatal appointments and the route of delivery. In Cluster 1, 72.1% (n=31) of births were vaginal and 27.9% (n=12) were cesarean. Similarly, Cluster 2 had 79.2% (n=19) vaginal births and 20.8% (n=5) cesarean. In contrast, Cluster 3 showed a markedly different pattern, with only 4.6% (n=2) vaginal births and 95.4% (n=41) cesarean sections.

The average number of emergency consultations in Cluster 2 was the lowest (3.5) compared to Cluster 1 (5) and Cluster 3 (5; *P*=.62).

**Table 2.** Comparison of patient clusters according to adherence to the app^a^^,^^b^.

Cluster	Control, n (%)	Nonadherent, n (%)	Adherent, n (%)
1	18 (32.7)	12 (35.3)	13 (61.9)
2	15 (27.3)	8 (23.5)	1 (4.8)
3	22 (40)	14 (41.2)	7 (33.3)
Total	55 (100)	34 (100)	21 (100)

a Chi-square test; significance set at *P*<.05.

b *P*=.12 for the overall comparison across the entire table.

### Clustering

In this study, clustering into three groups revealed significant differences in biopsychosocial aspects, which were named by the researchers according to the most striking characteristics of each group (Graph 2). Cluster 1—“experienced, disciplined mothers”—is characterized by multiparous women with lower incomes who had vaginal births, most of whom had more than one comorbidity, a longer history of deceased children, and good lifestyle habits, but who forget to take their medication and consider themselves less happy with their health. Cluster 2—“inexperienced pregnant women with limited involvement in care”—has the highest family income of the three clusters, with a predominance of women in their first pregnancy (nulliparous), with only one comorbidity and no deceased children; despite being the happiest with their health and considering the quality of their diet to be good, most of the patients here ignored what each medication was for, forgot to use their medication, and had the worst lifestyle habits, with higher alcohol consumption during pregnancy and worse quality of sleep. Cluster 3—“conscious multiparous”—is characterized by patients with more than one child, with high rates of cesarean sections, and mostly with only one comorbidity; these are the patients who best understand about the use of their medications and the ones who most remember to take them; although they sleep better than others, their diet is the worst of all clusters.

## Discussion

### Health Awareness

An app specifically designed for antenatal preparation in high-risk pregnancies has shown significant potential for enhancing health awareness and improving perinatal outcomes. Sakamoto et al [7] regard mHealth as an effective perinatal tool due to its cost-effectiveness and broad availability, as well as its capacity to empower pregnant women to manage their own health, promote healthy lifestyles, and access information anytime, anywhere. In our study, most patients brought their pregnancy-related test results to their antenatal appointments, although approximately 14.3% (n=8) of the control group and 9.1% (n=5) of the app group did not, suggesting gaps in patient education or communication at primary health units. Additionally, a portion of patients was unaware of the purpose of these tests, indicating potential shortcomings in the information provided to them during routine prenatal care.

The question “Do you consider yourself self-aware about your health?” highlighted that a minority of participants (n=7, 12.5%) in the control group and 14.5% (n=8) in the app group lacked a clear understanding of their health conditions. However, most patients consider preventive care to be an important aspect of health (n=54, 96.4% of the control group and n=55, 100% of the app group). DesJarlais [27] showed that, according to the Health Belief Model, women are more likely to seek preventive care if they believe that they are at risk and that the measures will bring more benefits than disadvantages. Many women are unaware of the benefits of antenatal care or have negative attitudes toward it, which may discourage them from seeking it even when it is available. Thus, instructing women on the importance of antenatal care is an important step to reduce health disparities [27], and mHealth proves to be a useful tool to increase women’s confidence and ability to make informed decisions about their care [28].

### Health Apps

Studies indicate that women’s health awareness, particularly during pregnancy, can significantly impact the overall health and well-being of both mother and baby, and that mHealth apps play a vital role in this period [29]. Women who have faced a clinical issue during pregnancy tend to increase their focus on health. Rossiter et al [30] observed that pregnant women’s motivation to adopt healthier behaviors was driven by the desire to avoid future health problems and improve overall well-being. The use of mHealth interventions, such as a health app for appointment preparation, consistently improves patients’ self-efficacy, providing tools for self-monitoring and self-care and improving health outcomes [7,29,31,32].

Adherence to an app depends on factors such as perceived usefulness, ease of use, performance, social influence, motivation, trust, privacy and security concerns, technology anxiety, personalization, and support [33-35]. Tailored content, a user-friendly interface, and technical stability improve experience and adherence [36], while demonstrated health benefits further drive adoption [37]. Conversely, technical issues, privacy worries, waning interest, and time constraints are primary barriers to continued engagement [34,36,38]. Addressing these can markedly enhance effectiveness [34].

Health professionals also play an important role by fostering awareness and motivation, providing credibility when endorsing apps [38], and reinforcing perceived usefulness and intent to use [39]. Through open dialogue, physical, and psychosocial support, especially in high-risk pregnancies, they help prevent distorted risk perceptions and ensure patients accept guidance and feedback during antenatal care [40-42]. Ultimately, adopting a person-centered approach through digital health apps such as the Health Assistant app offers promising opportunities to actively involve patients and families in antenatal care, leading to improved health care experiences and outcomes [6].

### App Usability: MAUQ

In this study, the Health Assistant app demonstrated good usability among high-risk pregnant women, as indicated by an average MAUQ score of 6.2 from the 23 users who engaged with the app. This result highlights the app’s ease of use and its perceived usefulness for antenatal preparation. Few studies have used the MAUQ for mHealth in pregnant women, and none of them in Brazil. A study conducted in Indonesia with 88 pregnant women to evaluate an app during pregnancy obtained an average MAUQ score of 6.26 in areas with a mobile signal, while in areas without a signal, the average was 6.29, which is similar to our finding [43].

Given that usability significantly impacts the adoption and sustained use of mHealth apps, particularly among populations with varying levels of health literacy, the positive usability scores from our participants suggest that the Health Assistant app effectively meets users’ expectations. Ensuring user-friendly interfaces and intuitive navigation remains essential in maximizing the potential benefits of digital health interventions in antenatal care. According to the study of the development and validation study of the original MAUQ, the higher the score, the greater the usability of the app [20]. Therefore, the average score of 6.2 obtained in this study is consistent with good usability.

### Clustering

The cluster analysis conducted in this study identified three distinct groups of high-risk pregnant women, each with specific biopsychosocial characteristics. This classification provided insights into behavioral and clinical patterns that may influence pregnancy outcomes and engagement with digital health interventions, detailed in the following sections.

Cluster 1, referred to as “experienced, disciplined mothers,” included mostly multiparous women with lower income and a history of multiple comorbidities. Although they reported healthier lifestyle habits and demonstrated the highest adherence to the Health Assistant app, they also showed a tendency to forget medication and reported lower satisfaction with their health. These findings align with previous research, suggesting multiparous women are more proactive in managing their complex health needs due to accumulated experiences [7,30].

Cluster 2, “inexperienced pregnant women with limited involvement in care,” was composed primarily of first-time mothers with higher incomes and fewer comorbidities. Despite reporting high satisfaction with their health and good dietary habits, this group had the poorest adherence to the app, the most frequent forgetfulness in medication use, and the highest rates of alcohol consumption and sleep dissatisfaction. This discrepancy between perceived and actual health behaviors aligns with the Health Belief Model, suggesting lower perceived susceptibility due to lack of previous negative health experiences, leading to reduced engagement in preventive behaviors [27,42,44]. Contrary to findings by Lee et al [45], this study observed lower app adherence among first-time mothers, highlighting a need for targeted education and personalized digital health interventions to enhance awareness of risks and benefits [46].

Cluster 3, identified as “conscious multiparous women,” exhibited moderate app adherence, better medication management, and high rates of cesarean deliveries. These women demonstrated a pragmatic and consistent adherence to medical recommendations, reflecting heightened awareness from previous pregnancy experiences [2,47]. Despite their good medication adherence and understanding of treatments, they reported poorer dietary habits, suggesting opportunities for targeted nutritional interventions.

Recognizing these distinct cluster profiles enables the development of tailored interventions to address the unique needs and challenges of each group, ultimately improving maternal-fetal outcomes and optimizing the use of mHealth tools in antenatal care. The main differences among the clusters are related to sociodemographic factors, obstetric history, and patient behavior. A detailed analysis of these aspects is essential for designing personalized strategies in education, adherence, and psychosocial support.

### Family Income

During the study period, the minimum wage in Brazil corresponded to approximately 260 dollars [48,49]. Patients with higher incomes and a better understanding of antenatal care may require less intensive interventions. In contrast, those from groups characterized by lower incomes and higher prevalence of comorbidities often need closer monitoring and additional support.

Cluster 2 stood out as all participants reported an income equal to or greater than two minimum wages. Conversely, Cluster 1 showed a significantly higher proportion of patients with lower income (n=14, 32.6% had incomes below two minimum wages). Interestingly, despite previous evidence suggesting that pregnant women from remote areas or lower socioeconomic backgrounds might experience difficulties accessing or effectively using mHealth technologies, our findings indicated the opposite [50]. We observed greater engagement with the app, precisely among participants with lower incomes (Cluster 1). This unexpected finding could be explained by the higher prevalence of comorbidities among these patients, motivating them to seek more active participation in their antenatal care. Additionally, the high proportion of multiparous women in this group (n=37, 86%) might indicate greater health awareness resulting from prior pregnancy experiences, further explaining their increased adherence [51].

### Medical History and Medication Adherence

In this study, multiparous women predominated in Clusters 1 and 3, while nulliparous women were the majority in Cluster 2. First-time pregnant women usually experience heightened awareness of bodily changes, possibly due to the novelty and uncertainty associated with pregnancy [52]. In this context, mHealth apps can serve as important tools, offering ongoing support and reassurance regardless of parity. Multiparous women, in contrast, often have better psychosocial adaptation during pregnancy and a greater appreciation for organization and planning based on previous experiences. This previous knowledge might explain their higher engagement with mHealth interventions observed in our results. Specifically, multiparous participants demonstrated greater adherence to the app, likely due to their familiarity with pregnancy-related risks and a proactive approach shaped by prior pregnancies. Thus, in our high-risk pregnancy context, previous experiences seem to strongly influence patients’ adherence, highlighting the importance of addressing both the informational needs of first-time mothers and reinforcing engagement strategies tailored for women with prior pregnancies [51].

### Previous Losses and Medication Adherence

The group consisting predominantly of first-time mothers with limited involvement in care (Cluster 2) stood out because none of these patients reported previous child losses. These women were characterized by fewer comorbidities (n=20, 83.3%) with only one comorbidity, possibly reflecting better overall health conditions. This may explain their lower adherence to the app, since fewer negative health experiences could lead to a reduced perceived need for additional monitoring and intervention [44]. Conversely, experienced mothers with disciplined engagement (Cluster 1) reported a higher incidence of having lost a child (n=10, 23.3%). This group’s socioeconomic profile—with lower income, a higher number of children, and more frequent comorbidities—likely contributed to these experiences. Such adverse life events might have fostered greater health awareness, thus motivating higher adherence to the mHealth intervention observed in this group.

Medication adherence is notably challenging among patients with chronic conditions, particularly in high-risk pregnancies [53]. Our findings demonstrated strong adherence among patients from Cluster 3, who all clearly understood the purpose of each medication prescribed. The fact that these patients were multiparous and had previously undergone cesarean sections further indicates heightened health awareness. This awareness translated into practical adherence behaviors, with the majority of these patients (n=32, 86.5%; *P*=.03) consistently taking their medications without forgetting. Understanding their medical conditions and the intended benefits of treatments clearly improved medication adherence in this group [6,54].

### Self-Awareness and Medication Management

Cluster 2 exhibited the lowest level of awareness regarding medication use, with 20% (n=4) of patients reporting uncertainty about the purpose of each medication. This was unexpected, given that this group was composed mostly of first-time pregnant women with higher income and fewer comorbidities—typically factors associated with greater engagement in health management. Conversely, Cluster 1 had the highest percentage (n=15, 40.5%) of patients forgetting to take medications. This vulnerability can be explained by the combination of lower income, higher number of children, and increased comorbidities observed in this group, resulting in more complex daily health demands and possibly less family or social support [55,56].

Although the app used in this study did not provide medication reminders, patients who actively used the app generally showed greater health engagement [28]. Apps have been recognized for their potential to facilitate medication adherence by increasing patient awareness and organization [28]. In this study, Cluster 1 patients were most engaged with the app; however, longer follow-up would be needed to determine whether app use directly influenced improved medication adherence over time. Given these observations, both Clusters 1 and 2 might benefit significantly from incorporating targeted medication adherence features into mHealth interventions [57,58].

### Lifestyle Habits

Adopting healthy lifestyle habits, such as regular physical activity, balanced nutrition, and adequate sleep, is highly recommended, particularly in high-risk pregnancies [59,60]. In this study, we identified differences in alcohol consumption between clusters. Cluster 2 (inexperienced pregnant women with limited involvement in care) presented the highest prevalence of alcohol use during pregnancy (n=4, 16.7%). This finding was unexpected since women with higher socioeconomic status generally demonstrate fewer risk behaviors during pregnancy [44]. Digital interventions, such as mHealth apps, have been shown to effectively reduce alcohol consumption in pregnant populations [44]. Therefore, this cluster could benefit significantly from increased engagement with such interventions.

Another notable aspect was sleep quality. Half of the patients in Cluster 2 reported inadequate sleep (*P*<.001). Enhanced app engagement might benefit these women, as mHealth-based interventions have previously demonstrated the ability to improve sleep quality and duration during pregnancy, positively influencing maternal health outcomes [61]. Diet quality perceptions varied among clusters. Interestingly, all patients in Cluster 2 rated their diet quality positively, whereas Cluster 3 was more critical (n=35, 81.4% considered it good; *P*=.006). It is possible that patients in Cluster 3, who displayed greater awareness and realistic views about medication use, similarly had more accurate self-assessments regarding their dietary habits.

We also observed significant differences between clusters in self-perceived happiness regarding health. Cluster 2 showed the highest proportion of women feeling happy with their health (n=18, 75%), whereas Cluster 1 had the lowest (n=16, 37.2%). This difference may be explained by the higher proportion of multiparous women in Cluster 1, whose additional life stressors and caregiving demands could negatively impact perceived happiness [62]. Additionally, the inherent challenges of high-risk pregnancies might further influence women’s emotional state and perceptions of happiness. Despite such challenges, previous research indicates that positive emotional states, such as happiness, can persist during high-risk pregnancies, particularly when reinforced by strong family support and personal resilience.

### Clusters, Outcomes, and Health Strategies

Identifying patient clusters allows targeted strategies to prevent adverse outcomes. Our findings revealed notable differences between groups regarding antenatal appointment attendance, route of delivery, and health behaviors.

The number of antenatal appointments varied significantly across clusters. Cluster 2 (“inexperienced pregnant women with limited involvement in care”) attended fewer appointments (6.9) compared to Clusters 1 (10.3) and 3 (9.1). This frequency falls short of World Health Organization (WHO) recommendations (minimum 8 visits), highlighting the need for intensified early follow-up and educational interventions, particularly considering their low adherence to the app [56,63]. Conversely, Cluster 1 attended the most appointments, likely due to greater medical complexity and higher app engagement [64]. Although the app did not send reminders, evidence from previous studies supports that increased mHealth use correlates with higher antenatal attendance, suggesting these groups could benefit from enhanced digital interventions [65]. It is noteworthy that, although statistically insignificant, Cluster 2 had fewer emergency department visits (3.5 visits). This can be related to the higher satisfaction with health in Cluster 2, but it can also have to do with higher income, which suggests that these patients, while receiving some care in the private sector, are perhaps keeping less frequent appointments only to retain a link with the public health system for childbirth.

Regarding delivery mode, Cluster 3 had significantly more cesarean sections (n=41, 95.3%) compared to Clusters 1 (n=12, 27.9%) and 2 (n=5, 20.8%; *P*<.001). This was likely due to the medical complexity of these multiparous patients and previous cesarean histories rather than mere personal preference [66,67]. Reducing unnecessary C-sections remains essential; thus, targeted education through the app could encourage safer delivery alternatives.

Pregnancy itself is a stimulus for lifestyle changes and positive health interventions [60], but cluster-based management strategies implemented by health teams could substantially improve pregnancy outcomes. Cluster 1**,** characterized by multiparity, low income, and high comorbidity, would benefit from interventions focusing on medication adherence, dietary optimization, and family planning guidance. Financial pressures in this group highlight the potential of mHealth tools to support self-management and health education [11]. It would also be interesting to enter information about these needs in the Health Assistant, as this was the group that adhered most to the app [68]. This would be in line with the findings of Wang et al [69], according to which patients expressed the need for apps containing evidence-based, personalized information to support them during pregnancy.

Cluster 2, despite higher socioeconomic status and fewer comorbidities, exhibited poor health behaviors such as alcohol use, inadequate sleep, and limited health awareness. Strategies to enhance app adherence, including personalized information and notifications, could significantly improve self-care and antenatal attendance. Integrating psychological support and mental health assessments into antenatal care can also address emotional well-being, increase self-awareness and self-care, and reduce risky behaviors [29].

Cluster 3, multiparous health-conscious women, demonstrated moderate app adherence (n=7, 33.3%) but lower self-rated diet quality. Targeted educational campaigns on nutrition, lifestyle, and informed choices regarding delivery methods could enhance patient engagement, improve maternal-fetal outcomes, and reduce unnecessary interventions [59]. Strategies for this group with moderate adherence to the app (n=7, 33.3%) could include educational campaigns to raise awareness about the benefits of the app, provide additional support to encourage its use, and increase attendance at antenatal appointments [70,71].

The cluster analysis presented in this study offers prenatal care teams a practical framework for stratifying pregnant patients according to their biopsychosocial risk profiles. By distinguishing groups with low engagement in antenatal care (Cluster 2) or difficulties in medication adherence (Cluster 1), health care professionals can more effectively identify individuals at heightened risk for disengagement or adverse pregnancy outcomes. Early recognition of these specific risk profiles enables teams to implement targeted, evidence-based interventions—such as personalized education, adherence support, and psychosocial assistance—before complications arise. This cluster-based approach facilitates efficient allocation of resources and proactive management, supporting improved maternal-fetal outcomes [6]. Furthermore, integrating mHealth solutions tailored to each cluster’s unique needs can enhance patient engagement and self-management, ultimately strengthening the quality and continuity of antenatal care.

### Implications for Future Research: “Clusters in Practice: A Proposal for a Risk Assessment Questionnaire for the First Antenatal Visit”

Based on the questions that most clearly differentiated our clusters, we propose developing a brief screening questionnaire to be administered at the first antenatal visit. This questionnaire would automatically indicate the most likely cluster for each patient, enabling rapid and specific interventions at the outset of prenatal care, thus preventing risks before undesirable behaviors or complications occur. Subsequent steps would involve drafting pilot items covering income, parity, comorbidities, and mHealth use profiles, submitting them to expert review and cognitive interviews with pregnant women for clarity and relevance, conducting a pilot study to assess construct validity (via factor analysis), internal consistency (Cronbach α), and test-retest reliability, and performing a prospective multicenter validation across diverse clinical settings to compare predicted risk scores with actual app engagement, appointment frequency, and maternal-fetal outcomes for sensitivity, specificity, and likelihood ratio estimation. Once validated, an algorithm could be embedded in eHealth records to assign each patient to a cluster and automatically trigger tailored interventions (eg, telemonitoring, reinforced health education, and SMS text message reminders), culminating in a randomized controlled trial contrasting “cluster‐guided” care with usual care using primary end points such as antenatal adherence rates, complication incidence, and health care resource utilization—an approach that, while consistent with established predictive‐health models, will require rigorous clinical validation and user‐acceptance testing to ensure accuracy, usability, and effectiveness before widespread implementation.

### Limitations

Though this study has provided us with several insights, it does have some limitations. Many patients were lost during data collection, which is often expected in observational longitudinal studies, but this loss, especially among prospective app users, can make it difficult to assess accurately the effectiveness of the intervention, since part of the target group was not thoroughly followed up. Since the transition from physical to digital records is still in progress, it is not uncommon for patients to resist mHealth adherence at first, and sample reduction in the adherent group in this study seems to have been due to such barriers as low literacy, limited connectivity, low smartphone memory, cultural beliefs, and lack of interest.

The fact that this study was carried out in a public university hospital in southern Brazil may render some generalizations invalid for other regions of the country or for private institutions. The adoption of three clusters yielded less variability in answers and more generalized results. Some sociodemographic factors overlapped among clusters, making it difficult for groups to be clearly distinguished and confusing for outcomes to be analyzed. In addition, the duration of the study, restricted to the period between the first antenatal visit and delivery, may not be sufficient to assess the long-term impacts of app use on maternal-fetal health and the well-being of pregnant women, thus indicating the need for further studies with larger samples and in different socioeconomic contexts.

### Conclusions

The use of mobile apps has significant potential to optimize antenatal care and self-awareness for high-risk pregnant women. The usability of the Health Assistant app is in line with the standards expected and observed in other studies. Cluster analysis revealed different profiles of pregnant women, making it possible to identify specific groups that could benefit most from digital interventions and from a personalized approach to organizing their journey and improving pregnancy outcomes. This pioneering study stresses the role of mHealth in high-risk antenatal care and highlights the importance of further exploration of its impact in different settings and populations.

## Abbreviations

MAUQ: Mobile App Usability Questionnaire
mHealth: mobile health
WHO: World Health Organization
